# Analysis of screening for neonatal hypoglycemia in large-for-gestational-age newborns without risk factors, and proposed changes in practice at Grenoble University Hospital

**DOI:** 10.18332/ejm/174489

**Published:** 2023-12-08

**Authors:** Marina Tamborowski, Sonia Ghelfi Dufournet, Lucie Terrier, Pierre Gillois, Lionel Di Marco

**Affiliations:** 1 Department of Midwifery, Faculty of Medicine, Grenoble Alpes University, Grenoble, France; 2 Department of Obstetrics, Couple Child Hospital, Grenoble Alpes University Hospital, Grenoble, France; 3 French National Centre for Scientific Research, Grenoble Alpes University Hospital Grenoble, France

**Keywords:** newborn, hypoglycemia, large-for-gestational-age, birth weight percentile

## Abstract

**INTRODUCTION:**

The aim of this study was to evaluate the relevance of screening for neonatal hypoglycemia as it is currently performed, in order to improve the comfort of newborns by reducing the number of painful procedures such as venipunctures or capillary punctures. The primary objective was to determine the prevalence of neonatal hypoglycemia in large-for-gestational-age newborns. The secondary objective was to determine a threshold percentile of birth weight for optimal screening for hypoglycemia.

**METHODS:**

We performed a descriptive, cross-sectional, single-center study, based on a structured review of obstetrical records from 11 January 2017 to 21 January 2020, from the maternity department of the University Hospital of Grenoble. Eligible neonates were large-for-gestational-age (birth weight >90th percentile) at term (37–42 weeks) without other risk factors for hypoglycemia. The primary outcome was the prevalence of neonates with capillary or venous glucose levels <2.2 mmol/L in the first 48 hours of life. We performed a sensitivity and specificity analysis of the birth weight percentile as a determinant of the threshold for hypoglycemia detection (ROC curve, area under the curve, Youden index, Brier score, Hosmer-Lemeshow test).

**RESULTS:**

In all, 19.2% of the newborns presented at least one hypoglycemic episode during the first 48 hours of life, and 75.7% of the hypoglycemic episodes occurred at 1 hour of life. The cut-off percentile that seemed most appropriate for screening was determined to be the 97th percentile of birth weight (AUC=0.64; 95% CI: 0.52–0.75).

**CONCLUSIONS:**

Our statistical model is robust and allows us to state that the currently used birth weight percentile threshold can be revised upwards. Thus, the protocol for neonatal hypoglycemia screening can be updated to improve the comfort of newborns at risk of hypoglycemia.

## INTRODUCTION

In 2016 in France, 6.8% of newborns were considered large-for-gestational-age (LGA) according to their birth weight percentile^1^. The definition of LGA newborn (‘macrosomic newborn’ in France) has varied over time, initially based on weight (4000 g) and more recently on the percentile corresponding to the birth weight for gestational age on a reference curve (90th percentile)^2,3^. In order to prevent metabolic complications^4^, newborns considered as LGA benefit from regular monitoring of capillary blood glucose levels (by minimally invasive sampling), which remains a painful procedure for them^5,6^.

Neonatal hypoglycemia is most often transient (within the first 48 hours of life)^7^; is considered to be early in the first 48 hours of life ; and presents a variety of clinical signs^8^. The diagnostic threshold for hypoglycemia is debated^9^, with a level <2.6 mmol/L being the most commonly used^10^, while other teams use a threshold value of 2.2 mmol/L^11^. The recommendations vary from one country to another, and in France there is currently no clinical practice guidelines on this subject.

In our daily practice, and in an empirical way, our view was that our protocol of screening for hypoglycemia in LGA newborns, without other risk factors, only rarely allowed the discovery of neonatal hypoglycemia. It seemed to us legitimate to question the realization of this painful procedure of little diagnostic relevance. We wondered whether considering a weight over the 90th percentile as a risk factor for hypoglycemia, was too broad a threshold, resulting in discomfort for monitored neonates when the benefit in terms of avoided hypoglycemia is potentially small. We therefore conducted a study at the request of the maternity department. To do this, we needed first to determine the true prevalence of hypoglycemia in LGA newborns without risk factors, then analyze the relevance of this test according to the birth weight percentile of the newborns.

The primary objective of this study was to determine the prevalence of hypoglycemia in term and LGA neonates, without other risk factors for hypoglycemia, currently classified as hypoglycemic episode risk at the Couple/Child Hospital (HCE in French) of the University Hospital of Grenoble. The secondary objectives were to assess the time of life at which newborns were most at risk of hypoglycemia and to extract a threshold percentile for blood glucose monitoring in LGA newborns.

## METHODS

We performed a descriptive, cross-sectional, single-center study, based on a structured review of obstetrical records from 11 January 2017 to 21 January 2020, from the maternity department (HCE) of the University Hospital of Grenoble. Eligible neonates were LGA (here a birth weight >90th percentile) at term (37–42 weeks) without other risk factors for hypoglycemia. The Table 1 shows the excluded newborns.

**Table 1:** Excluded newborns

*Exclusion criteria*
∙ Insulin-requiring mother (type 1 or 2 diabetes, insulin-requiring gestational diabetes)
∙ Mother with hypoglycemic treatment (diuretics, beta-blockers, anti-epileptics, corticoids),
∙ Mother taking toxic drugs
∙ Pregnancies with an unclear term or not followed up
∙ Risk of acute fetal suffering in the perinatal period
∙ Indications of neonatal transfer
∙ Twin pregnancies with transfusion-transfusion syndrome (TTS), twin anemia polycythemia sequence (TAPS), twin reversed arterial perfusion (TRAP) or twin oligohydramnios polyhydramnios sequence (TOPS)

The primary judgement criterion was the prevalence of LGA term newborns with proven hypoglycemia in the first 48 hours of life; hypoglycemia was defined as at least one capillary or venous blood glucose level <2.2 mmol/L in the first 48 hours of life according to the neonatal blood glucose monitoring protocol of the HCE. The protocol used in our maternity hospital designates the newborns to be screened (Table 2).

**Table 2:** Protocol of hypoglycemia screening in Grenoble-Alpes University Hospital

*Infants to be screened*
Premature infants aged <36 weeks
Birth weight ≥90th percentile
Birth weight <2500 g
Newborn of diabetic mother on insulin
Newborn of mother on beta-blockers
Acute fetal distress, respiratory distress
Newborn with abnormal clinical signs (tremors, cyanosis, convulsions, apnea)
***Monitoring carrying out***
At 1 hour of life before the first feeding, given no later than 2 hours of life
At 4 hours of life before the 2nd meal
Then before every second meal during the first 24 hours of life
Then before every fourth meal for the next 24 hours
At 1 hour of life before the first feeding, given no later than 2 hours of life

Then, 2 classic care-protocols are used for newborns with hypoglycemia. The ‘4-hour wake-up’ protocol refers to regular blood glucose monitoring in newborns who need to wake up or be woken up at least every 4 hours in order to be fed. The ‘3-hour wake-up’ protocol refers to regular blood glucose monitoring in neonates who need to wake up or be woken up at least every 3 hours in order to be fed.

The first judgment criterion was the description of the time of life at which newborns were most at risk of hypoglycemia, and, secondly, the sensitivity and specificity of the test for each percentile of birth weight. Finally, we determined a birth weight percentile that could be used as a new threshold for classifying LGA neonates as being ‘at risk for neonatal hypoglycemia’.

### Statistical analysis

Statistical analyses were performed using Statview^®^, XLSTAT^®^ and R^®^ software. To identify possible factors influencing neonatal hypoglycemia, we compared population characteristics using a chi-squared test. We used a ROC curve, its area under the curve, and the Hosmer-Lemeshow test that shows the difference between the model and the data, concerning the sensitivity and specificity. The Youden index was used to determine a birth weight percentile that could be used as a new threshold for classifying LGA neonates as being ‘at risk for neonatal hypoglycemia’. The performance of this test was evaluated by the Brier score.

## RESULTS

### Population characteristics

**Figure 1:**
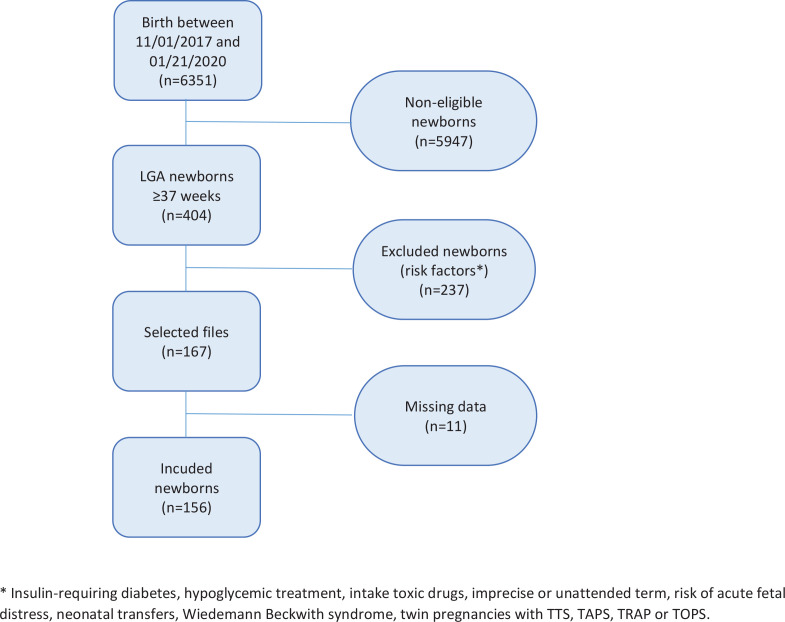
Flow chart of the 2017-2020 screening of neonatal hypoglycemia

Figure 1 shows that out of 6351 births, 404 concerned LGA newborns and 156 children were included (167 eligible including 11 missing data). Regarding maternal history, 37 mothers (23.7%) had a history of LGA or macrosomic children and 27 mothers (17.3%) had diet-induced gestational diabetes for this pregnancy.

In the sample included, 101 children followed the ‘4-hour wake-up’ protocol (64.7%), 1 followed the ‘3-hour wake-up’ protocol (0.3%), 2 followed a specific protocol decided by the pediatrician (1.3%), and 45 had no protocol indicated (28.8%).

The protocol requires a minimum number of blood glucose tests to be performed to monitor neonatal blood glucose and a maximum time between two blood glucose tests. However, this protocol was not perfectly adhered to. The minimum number of tests was met for 90 neonates (57.7%) while the maximum time between two blood glucose tests was met for 22 neonates (14.1%). In accordance with our objectives, and due to lack of data, the reasons for this non-compliance were not collected.

Among all the characteristics of the population studied, several criteria influencing the occurrence of early neonatal hypoglycemia were found: birth weight (χ²=7.72; p=0.021), birth weight percentile (χ²=8.39; p=0.015), history of LGA or macrosomia (χ²=12.43; p=0.002), history of pre-eclampsia (χ²=12.43; p=0.002), history of eclampsia (χ²=6.43; p=0.04), history of gestational diabetes (χ²=10.59; p=0.005), and route of delivery (χ²>18.42; p<0.0001).

### Primary objective

The prevalence of at least one hypoglycemia in the first 48 hours of life (with a protocol threshold of 2.2 mmol/L) in LGA newborns without risk factors was 19.2%, regardless of time of onset (37.2% if the threshold of 2.6 mmol/L was used).

### Secondary objectives

The median time of first hemoglucotest measurement was 1 hour of life with a median result of 2.9 mmol/L. A total of 25 hypoglycemic episodes (75.7%) occurred in the first hour of life and 2 in the sixth hour of life (6.1%). The other hypoglycemic episodes were later and occurred at 24, 34, 35, 38 and 44 hours of life.

It can be seen that as the number of measurements increases, the number of newborns monitored by hemoglucotest decreases: a clear break is observed from the sixth measurement onwards, with a median time of completion at 45 hours of life (Table 3).

**Table 3:** Specifications of screenings

*Measurements*	*Number (N=156) n (%)*	*Time (hours) median (IQR)*	*Value (mmol/L) median (IQR)*	Hypoglycemia (N=33)^*^ n (%)
1	155 (92.8)	1 (1–2)	2.9 (2.4–3.6)	25 (75.7)
2	151 (90.4)	5 (5–8)	3.4 (2.9–3.8)	2 (6.1)
3	149 (89.2)	14 (11–18)	3.6 (3.2–3.9)	1 (3.0)
4	144 (86.2)	22 (19–28)	3.7 (3.2–4.1)	0
5	128 (76.6)	34 (25–42)	3.6 (3.2–3.9)	2 (6.1)
6	90 (53.9)	45 (36–51)	3.8 (3.3–4.3)	2 (6.1)
7	39 (23.4)	51 (43–57)	3.8 (3.3–4.1)	0
Supplementary 1	13 (7.8)	53 (41–57)	3.4 (2.6–4.2)	0
Supplementary 2	8 (4.8)	62 (42–66)	3.4 (3.0–4.6)	1 (3.0)
Supplementary 3	4 (2.4)	48 (39–64)	3.1 (3.1–3.8)	0
Supplementary 4	2 (1.2)			0
Supplementary 5	1 (0.6)			0

IQR: interquartile range.

* 3 newborns presented 2 hypoglycemic episodes during the 48 first hours of life.

**Figure 2:**
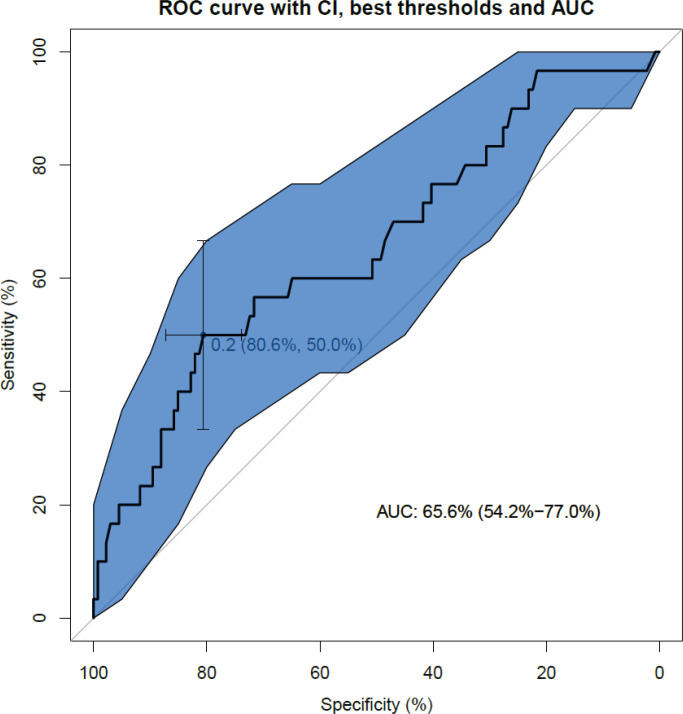
ROC curve of birth weight percentile as a determinant of the occurrence of early neonatal hypoglycemia

The ROC curve (Figure 2) analyzing the specificity and sensitivity of the birth weight percentile as a determinant of the occurrence of early neonatal hypoglycemia (occurring within the first hour of life) shows an area under the curve (AUC) of 0.64 (95% CI: 0.52–0.75). This AUC in Figure 2 shows a slightly informative discriminatory capacity as it is slightly higher than the dotted diagonal representing an AUC of 50%.

The ROC curve and the AUC value are validated by the Hosmer-Lemeshow test (p=0.29), which assesses whether or not the observed event rates correspond to the expected event rates in the model’s population subgroups. Thus, models for which the expected and observed event rates in the subgroups are similar are said to be well calibrated, as in our study.

The model’s performance was judged to be high based on the Brier score^12^ calculated at 0.15. This score takes a value between 0 and 1, since it is the square of the greatest possible difference between a predicted probability and the actual outcome.

Finally, the maximum Youden index determines the threshold percentile to be retained as the 97th percentile of birth weight. This index identifies the diagnostic test threshold that minimizes the sum of false-negatives and false-positives.

## DISCUSSION

Our study determined that the prevalence of at least one neonatal hypoglycemic episode was 19.2%, regardless of the time of onset. This appears to be a fairly low prevalence. But it should be noted that our definition of hypoglycemia, determined by the protocol used in our hospital, is particularly low (2.2 mmol/L instead of 2.5 or 2.6 mmol/L in the literature). Thus, we would rather approach 37% with a threshold more in line with the literature.

The median time of the first hemoglucotest measurement was 1 hour of life with a median result of 2.9 mmol/L. Neonatal blood glucose results from a balance between tissue glucose utilization, glycogenolysis and gluconeogenesis, and exogenous inputs^8^. Glycogenolysis ensures glucose production during the first 12 hours of life while gluconeogenesis starts after 3 hours of life. The concentration of lactate (substrate of cerebral energy metabolism) is maximal during the first 3 hours of life while the concentration of ketone bodies (supplier of alternative substrates) increases until reaching its nadir at 12 hours of life^13^ . Despite a rapid drop in blood glucose at birth to reach its nadir at 1 hour of life and a stabilization from the third hour^5,8,14,15^, this is compensated by the use of lactates and ketone bodies as a transient energy substrate. Thus, although currently counted in the prevalence of early neonatal hypoglycemia, hypoglycemia occurring in the first hour of life can be considered physiological^9^. This physiological hypoglycemia at 1 hour of life is confirmed in exclusively breastfed neonates: their glucose level is lower while the ketone body level is higher than neonates fed with artificial milk^14,15^ showing a positive adaptive response to this period of low food intake^16^.

Finally, the specificity and sensitivity analysis of the birth weight percentile as a threshold for hypoglycemia detection was 97. Although the AUC of our ROC curve is only slightly greater than 0.5 (AUC=0.64; 95% CI: 0.52–0.75), the robustness of our study was demonstrated by a Hosmer-Lemeshow test and a Brier score that were both significant. This confirms our initial hypothesis and allows us to start a local reflection on the comfort of newborns in the context of neonatal hypoglycemia screening. Perhaps it is necessary to set up a later paraclinical surveillance, by reinforcing the clinic during the first hour of life, as well as the prevention via an early feeding and a protocol better followed by the professionals.

### Strengths and limitations

Our work has fairly strong internal validity as shown by the Hosmer-Lemeshow test and the Brier score despite the AUC value. We were limited in our access to complete ultrasound reports, which did not allow us to distinguish between constitutional and overgrowth LGA. Thus, the prevalence of hypoglycemia in our population may be artificially low, but this would have little impact on our secondary objectives. While confounding bias was minimized by the literature review, some criteria that could generate hypoglycemia were identified a posteriori: history of LGA or macrosomia in a previous pregnancy, history of pre-eclampsia or eclampsia in a previous pregnancy, history of gestational diabetes without the occurrence of LGA, and route of delivery in the current pregnancy. Thus a multivariate analysis on a larger sample could allow these elements to be taken into account.

Concerning the external validity of our work, the literature (at identical thresholds) shows the same prevalence of neonatal hypoglycemia^11^.

We have already discussed this: although currently counted in the prevalence of early neonatal hypoglycemia, some authors consider hypoglycemia occurring within the first hour of life to be physiological^9^. In our sample, the prevalence of hypoglycemia occurring after 1 hour of life is only 5.3%, without prolonged hypoglycemia. Several recommendations have been made in this regard, including not starting screening before 3 hours of life (or the second feeding) and not continuing screening beyond 12 hours of life if a blood glucose level ≥2.6 mmol/L is maintained at the time of monitoring^9^.

The Brier score in our study provided no evidence of any clinical reality of the 90th percentile threshold for detection of neonatal hypoglycaemia^13^. However, we found a threshold at the 97th percentile of birth weight to have the best sensitivity/specificity ratio in predicting the occurrence of neonatal hypoglycemia. Our study is a starting point whose results need to be confirmed by a larger study but also with multivariate statistical analysis as several factors have a statistically significant influence on the occurrence of neonatal hypoglycemia in LGA newborns.

## CONCLUSIONS

This study allowed us to analyze our practice of screening for neonatal hypoglycemia in LGA neonates without other risk factors. The prevalence of hypoglycemia in our sample is comparable to that found in the literature. The robustness of our model, despite a low area under the curve, allows us to conclude that the threshold at the 97th percentile is still significant. The physiological hypoglycemia at 1 hour of life as well as the low prevalence of hypoglycemia after 1 hour of life, point to a possible excessive monitoring of neonatal hypoglycemia in LGA newborns, contributing to avoidable neonatal discomfort.

The challenge will be to extend this study to all LGA newborns, regardless of the number of risk factors for hypoglycemia. Consideration should also be given to reviewing professional practice (currently based solely on professional agreement) via larger studies of the association between birth weight percentile and the occurrence of early neonatal hypoglycemia, as our study has shown.

## Data Availability

The data supporting this research are available from the authors on reasonable request.
